# Oxidative Stress-Related Mechanisms and Antioxidant Therapy in Diabetic Retinopathy

**DOI:** 10.1155/2017/9702820

**Published:** 2017-02-06

**Authors:** Cheng Li, Xiao Miao, Fengsheng Li, Shudong Wang, Quan Liu, Yonggang Wang, Jian Sun

**Affiliations:** ^1^The First Hospital of Jilin University, Changchun 130021, China; ^2^The Second Hospital of Jilin University, Changchun 130041, China; ^3^General Hospital of the PLA Rocket Force, Beijing 100088, China

## Abstract

Diabetic retinopathy (DR) is one of the most common microvascular complications of diabetes and is the leading cause of blindness in young adults. Oxidative stress has been implicated as a critical cause of DR. Metabolic abnormalities induced by high-glucose levels are involved in the development of DR and appear to be influenced by oxidative stress. The imbalance between reactive oxygen species (ROS) production and the antioxidant defense system activates several oxidative stress-related mechanisms that promote the pathogenesis of DR. The damage caused by oxidative stress persists for a considerable time, even after the blood glucose concentration has returned to a normal level. Animal experiments have proved that the use of antioxidants is a beneficial therapeutic strategy for the treatment of DR, but more data are required from clinical trials. The aims of this review are to highlight the improvements to our understanding of the oxidative stress-related mechanisms underlying the development of DR and provide a summary of the main antioxidant therapy strategies used to treat the disease.

## 1. Introduction

Diabetes, a chronic metabolic disease, includes types I (lack of insulin) and II (insulin resistance). Globally, approximately 415 million people suffer from diabetes, and one person dies every six seconds with this disease. Moreover, a new patient is diagnosed every two seconds [1]. Diabetic retinopathy (DR), a sight-threatening microvasculature impairment, is one of the complications that seriously threaten the life of diabetic patients. DR is acknowledged as the main cause of blindness among working-age adults throughout the world [2]. In 2010, there were 126.6 million patients with DR, and it is predicted that this figure will increase to 191.0 million by 2030; consequently, the number of those with sight-threatening DR will increase from 37.3 million to 56.3 million during this period [3].

The development of DR is associated with sustained metabolic disorders caused by hyperglycemia and increased levels of inflammatory cytokines in the blood. Systemic inflammation caused by these metabolic alternations leads to hemodynamic changes, blood-retinal barrier (BRB) damage, the leakage of retinal microvessels and edema, a gradual thickening of the retinal vascular basement membrane, and a loss of pericytes. These lesions continue with the progression of diabetes. During the first few years of diabetes, patients may barely be aware of, or exhibit, retinal injury. However, the obvious symptoms of DR will be present in nearly all patients with type I diabetes for 20 years and in nearly 80 percent of those with type II diabetes for the same duration [4]. Therefore, an understanding of the mechanisms by which these pathogenic alterations are induced will improve the development of effective strategies to prevent and postpone the progression of DR.

Oxidative stress is thought to be one of the crucial factors in the pathogenesis of DR. Abnormal metabolism induced by hyperglycemia can result in the overproduction of free radicals such as hydroxyl and superoxide radicals, which are known as reactive oxygen species (ROS) [5]. The accumulation of ROS can lead to oxidative stress, which damages the tissue in and around retinal vessels, ultimately resulting in DR. It is well known that hyperglycemia can cause vascular damage through four classical mechanisms: increased polyol pathway flux; increased intracellular formation of advanced glycation end-products (AGEs) and expression of the receptor for AGEs; activation of the protein kinase C (PKC) pathway; and activation of the hexosamine pathway [6]. Moreover, the nuclear factor, erythroid 2 like 2- (NFE2L2-) related pathway (NFE2L2 is also known as Nrf2), GTP-binding proteins, and epigenetic modifications have also attracted increasing attention in recent years [7–9]. All these pathways are associated with the overproduction of ROS. In particular, oxidative stress induced by epigenetic modifications can persist for a considerable time, even after the blood glucose concentration returns to normal; this is called “metabolic memory” [10]. Thus, scavenging and/or reducing ROS production by these pathways may provide new therapeutic strategies. At present, multiple antioxidants have been used in clinical trials, but the results are not clear. In this review, we summarize several of the mechanisms induced by oxidative stress that cause DR and focus on the latest research into the treatment of the disease using antioxidants.

## 2. Diabetes-Induced Production of ROS

**Figure 1:**
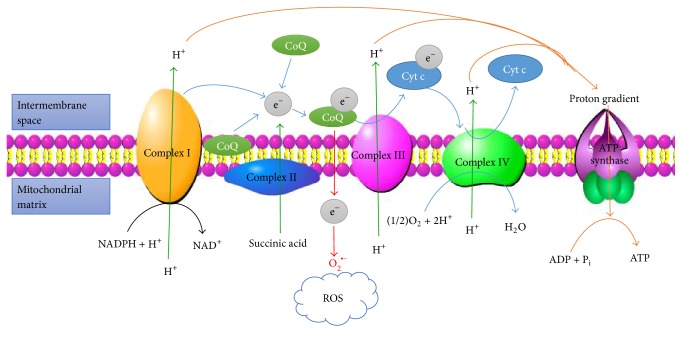
The electron transport in the mitochondrial respiratory chain and the production of ROS. Complexes I–IV are located in the mitochondrial inner membrane. First, complexes I and II can accept electrons from NADPH and succinic acid and then transport them to coenzyme Q (CoQ), while at the same time, they can pump out protons. Then, complex III can transfer electrons from CoQ to cytochrome C (Cyt c). Finally, complex IV sends electrons to O_2_, producing H_2_O. During this process, a proton gradient is formed, which promotes the synthesis of ATP. If complex III cannot receive electrons from CoQ, the electrons would be accepted by O_2_, which could produce ROS and result in oxidative stress.

Oxidative stress can induce damage to target organs such as the retina [11]. The production of ROS mainly depends on two factors: (a) mitochondrial oxidative phosphorylation [12] and (b) the nicotinamide adenine dinucleotide phosphate- (NADPH-) oxidase (Nox) system [13]. Mitochondria are the major endogenous source of ROS and can utilize 95% of the available oxygen to produce ATP (Figure 1). Normally, ~2% of oxygen enters the electron transport chain and is subsequently oxidized to superoxides such as O^2−^ and hydrogen peroxide. In diabetes, the uncoupling of mitochondrial electron transport leads to excessive superoxide production, which may stimulate several abnormal biochemical metabolic pathways, such as the polyol, PKC, and AGE pathways [14]. It has been reported that the levels of transcription of mitochondrial DNA-encoded NADH dehydrogenase 1 and 6 of complex I and cytochrome b of complex III are subnormal in the retinas of diabetic patients [15, 16], which contributes to the development of DR. Moreover, superoxides can also react with nitric oxide (NO) to form peroxynitrite. Peroxynitrite can oxidize small-molecule antioxidants such as glutathione (GSH), cysteine, and tetrahydrobiopterin [17], resulting in cytotoxicity due to lipid peroxidation, inactivation of enzymes by oxidation of sulfhydryl moieties and nitration of tyrosine, and damage to DNA [14, 18, 19].

The Nox system can generate ROS and catalyze molecular oxygen to produce superoxides and/or hydrogen peroxide by accepting electrons from NADPH and transporting them to molecular oxygen. The Nox system is a major source of oxidative stress in the vascular system and can be divided into different isoforms. Vascular Nox comprises membrane-bound subunits (Nox protein and p22phox) and cytosolic subunits (p47phox, p67phox, and Rac). Isoforms Nox 1, Nox 2, and Nox 4 are highly expressed in the vascular system [20]. The activity of Nox 2 increases in the retinas of diabetic mice. This increase is associated with increased production of ROS and the expression of intercellular adhesion molecule-1 (ICAM-1) and vascular endothelial growth factor (VEGF), which can be inhibited by deletion of the Nox 2 gene [21]. A recent study has shown that Nox 1 expression and activity increase in the microvascular endothelial cells in the brains of diabetic mice [22]. Similar findings have also been reported in kidney vessels, coronary microvessels, and aortic endothelial cells [23, 24].

## 3. Hyperglycemia-Induced Pathological Changes in DR

The retina is the most metabolically active tissue in the body and is therefore easily affected by diabetes. Sustained hyperglycemia damages the microvasculature of the retina resulting in hemodynamic changes, thickening of the basement membrane, loss of pericytes, and BRB dysfunction [25, 26]. These abnormalities cause retinal ischemia and the release of proinflammatory and proangiogenic factors, which lead to inflammation and angiogenesis. The tight junctions between retinal pigment epithelial (RPE) cells constitute the BRB, which protects the retina from abnormal effusion from the choroid. Hyperglycemia produces elevated levels of methylglyoxal due to increased glycolysis. Methylglyoxal activates matrix metalloproteinases, which may facilitate an increase in vascular permeability by a mechanism involving the proteolytic degradation of tight junction proteins such as occludin. Fluid leakage therefore increases in the surrounding retinal tissue, resulting in macular edema and visual loss [27].

The retina is susceptible to oxidative stress owing to its hypermetabolic state [28], and the levels of oxidative stress markers are related to the severity of DR [29]. Experiments have demonstrated that the degeneration of retinal capillaries in diabetes can be reduced by antioxidant therapy via activation of caspase-3 and nuclear factor-*κ*B (NF-*κ*B), indicating that oxidative stress plays an important role in retinal capillary apoptosis [30, 31]. The dysfunction of rod and cone photoreceptors is thought to contribute to the development of retinal hypoxia and neovascularization [32, 33]. Patients with diabetes and the outer retinal degenerative disorder retinitis pigmentosa have a reduced risk of the development of preproliferative DR [34, 35]. It has been demonstrated that the retinas of rhodopsin knockout mice exhibit vascular attenuation in the capillary bed; however, in the retina of rhodopsin knockout mice with diabetes, it appears to have fewer vascular attenuation than that of nondiabetic counterparts [36]. The reason may be that the loss of rod photoreceptors during retinitis pigmentosa leads to a net reduction in oxygen usage by the retina. Thus, it may offset the exacerbation of hypoxia during DR, thereby protecting the microvasculature from pathogenic change. Furthermore, hyperglycemia-induced endothelial injury can produce reactive nitrogen species through the activation of arginase, leading to disruption of the nitroso-redox balance [37]. Finally, increased levels of nitrogen species, including NO and peroxynitrite, promote leukocyte adhesion to retinal vessels, BRB breakdown, and RPE damage [38, 39].

## 4. Role of Oxidative Stress in Hyperglycemia-Induced Pathological Changes in DR

### 4.1. General Mechanisms Underlying DR

**Figure 2:**
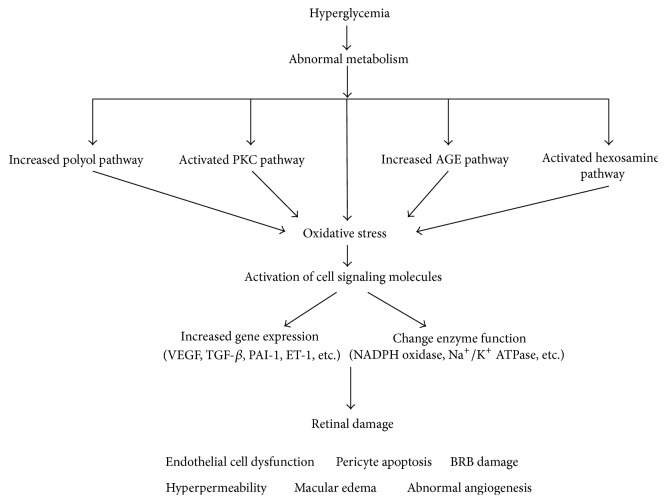
The mechanisms of oxidative stress and diabetic retinopathy. BRB, blood-retinal barrier; ET-1, endothelin-1; NADPH, nicotinamide adenine dinnucleotide phosphate; PAI-1, plasminogen activator inhibitor 1; TGF-*β*, transforming growth factor-*β*; VEGF, vascular endothelial growth factor.

As shown in Figure 2, four classical mechanisms are involved in the pathology of DR: increased polyol pathway flux, PKC pathway activity, accumulation of AGEs, and activation of the hexosamine pathway.

Hyperglycemia induces the activation of the polyol pathway and increases intracellular glucose flux [40]. Excess intracellular glucose is converted to sorbitol by aldose reductase, and sorbitol is oxidized to fructose by sorbitol dehydrogenase. During the reaction, these two enzymes can oxidize NADPH to NADP^+^and convert NAD^+^ to NADH, respectively. As a result, NADPH is consumed, the ratio of NADH/NAD^+^ increases, and the synthesis of NO and GSH decreases, all of which are important to maintaining the redox balance. Moreover, the accumulation of sorbitol is associated with basement membrane thickening, endothelial cell death, and pericyte apoptosis [41, 42]. Thus, activation of the polyol pathway plays a crucial role in the pathogenesis of DR.

**Figure 3:**
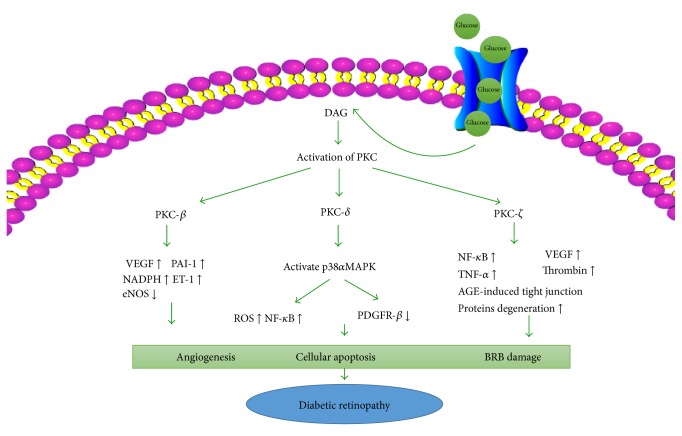
The activation of the three main protein kinase C (PKC) isoforms induced by hyperglycemia. AGE, advanced glycation end-products; BRB, blood-retinal barrier; DAG, diacylglycerol; eNOS, endothelial nitric oxide synthase; ET-1, endothelin-1; NADPH, nicotinamide adenine dinnucleotide phosphate; NF-*κ*B, nuclear factor-*κ*B; PAI-1, plasminogen activator inhibitor-1; PDGFR, platelet-derived growth factor receptor-*β*; TNF-*α*, tumor necrosis factor-*α*; VEGF, vascular endothelial growth factor.

PKC is a serine/threonine-related protein kinase; it mainly has three isoforms that are involved in diabetes: PKC-*β*, PKC-*δ*, and PKC-*ζ*. Hyperglycemia primarily activates PKC-*β*, which is associated with neovascularization. PKC-*β* can increase the expression of VEGF [43]. Retinal ischemia induces the overexpression of PKC-*β* in transgenic mice, whereas a lack of PKC-*β* reduces angiogenesis [44]. PKC-*δ* can be activated by aldose reductase [45], and the PKC-*δ*/p38*α*MAPK pathway can inhibit platelet-derived growth factor-mediated survival activity, resulting in the apoptosis of pericytes and the formation of acellular capillaries [46]. PKC-*ζ* can be detected in endothelial cells and is involved in VEGF-mediated proliferation and hyperpermeability induced by tumor necrosis factor-*α* (TNF-*α*) and thrombin (Figure 3) [47, 48].

AGEs are the late products of nonenzymatic glycation. Normally, glucose forms a Schiff base through the Maillard reaction; irreversible oxidation and dehydration reactions then convert Amadori products to AGEs [49]. Oxidative stress causes the formation of reactive carbonyl compounds, which react with protein, resulting in the production and accumulation of AGEs [50]. AGEs can cross-link with structural proteins such as elastin, laminin, and collagen type IV, leading to reduced elasticity and sensitivity to proteolytic digestion [51]. In addition, the interaction between AGEs and their cell surface receptor mediates the production of ROS, the activation of NF-*κ*B [52], and the expression of VEGF, monocyte chemoattractant protein-1, and plasminogen activator inhibitor-1. This contributes to hyperpermeability and the breakdown of the BRB, ultimately leading to angiogenesis and thrombosis [53, 54].

Increased levels of ROS under hyperglycemic conditions inhibit the activity of glyceraldehyde-3-phosphate dehydrogenase, which diverts fructose 6-phosphate from the glycolysis pathway to the hexosamine pathway, thereby activating the hexosamine pathway and producing UDP-N-acetylglucosamine [55]. UDP-N-acetylglucosamine is an important substrate in the posttranslational modification of Ser/Thr residues by O-linked *β*-N-acetylglucosamine. A recent study demonstrated that hyperglycemia promotes the GlcNAcylation of NF-*κ*B, which contributes to the death of retinal gangliocytes [56]. Moreover, elevated angiopoietin-2 (Ang-2) levels cause pericyte loss, which is probably related to the increased GlcNAcylation of stimulating protein-3 (Sp-3) through the promotion of Sp-1 binding to the Ang-2 promoter and activation of the transcription of Ang-2 [57].

### 4.2. Nrf2-Keap1 Antioxidant Defense System in DR

**Figure 4:**
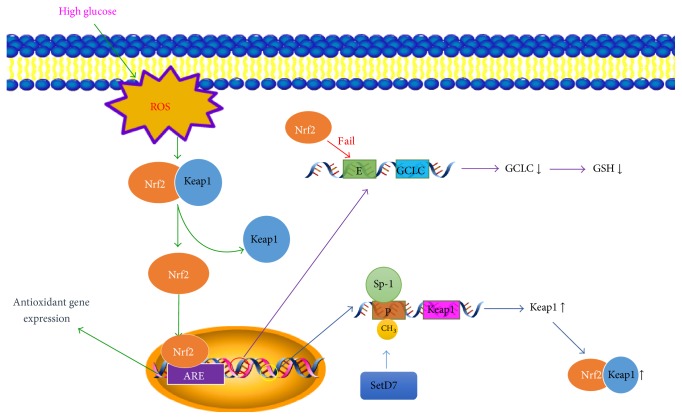
Nrf2-related signal transduction. Oxidative stress activates Nrf2 via dissociating it from its inhibitor Keap1 and translocating to the nucleus, where it binds with the antioxidant-response element (ARE) to regulate transcription of antioxidant genes. At the same time, the expression levels of two other genes are also influenced. On the one hand, Nrf2 fails to bind to the enhancer region of glutamate cysteine ligase catalytic subunit (GCLC), which is an important enzyme in glutathione (GSH) synthesis, thus resulting in the decrease of GSH. On the other hand, hyperglycemia facilities methylation of the Keap1 promoter via methyltransferase enzyme Set7/9 (SetD7), which eases stimulating protein-1 (Sp-1) binding to the promoter, resulting in increased Keap1 expression; therefore, Keap1 combines with Nrf2 and represses Nrf2 in the cytosol. ARE, antioxidant-response element; CH_3_, methylation; E, enhancer; GCLC, glutamate cysteine ligase catalytic subunit; GSH, glutathione; P, promoter; SetD7, methyltransferase enzyme Set7/9; Sp-1, stimulating protein-1.

Nrf2 is a redox-sensitive factor that can regulate the transcription of antioxidant genes and act as a protective factor in inflammation and ischemia/reperfusion injury [58, 59]. Normally, Nrf2 exists in the cytosol and binds to its cytosolic inhibitor Kelch-like ECH-associated protein 1 (Keap1). When oxidative stress occurs, Nrf2 dissociates from Keap1 and translocates to the nucleus, where it binds with the antioxidant-response element (ARE) to regulate the transcription of antioxidant genes (Figure 4) [7]. Thus, the Nrf2-Keap1-ARE pathway is considered one of the major ways by which cells are protected from oxidative stress, and it can regulate the expression of a number of protective factors, such as GSH [7, 60]. GSH biosynthesis can be regulated by glutamate cysteine ligase (GCL), which has a catalytic subunit (GCLC) and a modifier subunit (GCLM). The transcription of GCLC can be regulated by Nrf2 [7]. It has been confirmed that Nrf2 is activated in streptozotocin- (STZ-) induced mouse retinas, and the level of nuclear Nrf2 in diabetic mice is two times higher than in nondiabetic controls. Moreover, in Nrf2-deficient diabetic mice, the levels of ROS, TNF-*α*, and superoxide increase, and the level of GSH decreases [7, 61]. Under high-glucose conditions, there is a decrease of Nrf2 binding at the antioxidant-response element region 4 (ARE4), leading to the decreased expression of its downstream target gene* GCLC*. Moreover, histone methylation at GCLC-ARE4 can disrupt the binding of Nrf2 with GCLC-ARE4, which is not altered by the termination of high-glucose insult [62]. This suggests an important role of the Nrf2-Keap1-GCLC-GSH signaling pathway in the maintenance of retinal redox status in the development of DR.

Epigenetic modifications of Keap1 also play an important role in the development of DR. Keap1 is a crucial factor during Nrf2 signal transduction because it is a substrate adaptor protein for Cullin3/Rbx1 ubiquitin ligase. The Keap1 promoter has an Sp-1 element site, and the binding of Sp-1 with the Keap1 promoter can activate the transcription of Keap1 [63]. Hyperglycemia can activate methyltransferase enzyme Set7/9 (SetD7), which facilitates the methylation of lysine 4 on histone 3, and increases the binding of Sp-1 to the Keap1 promoter. Moreover, SetD7-siRNA decreases the binding of Sp-1 to the Keap1 promoter and reduces its expression; thus, the functions of Nrf2 are suppressed. Termination of a high-glucose insult fails to reduce the binding of Sp-1 to Keap1, and the methylation of the promoter persists with the increased expression of Keap1, which implies that it contributes to “metabolic memory” [64]. A recent study has shown that NF-*κ*B activity is enhanced by reduction of the deacetylase activity of sirtuin 1 (SIRT1) by high-glucose levels, which leads to downregulation of microRNA-29 (miRNA-29) and inhibition of Keap1/Nrf2 signaling [65]. Accumulating evidence supports the protective role of Keap1 in various cancers via regulation by miRNAs such as miRNA-200a and miRNA-141 [66, 67], although studies on DR are scarce. This suggests that miRNAs may have therapeutic potential for the clinical treatment of DR.

### 4.3. Novel Small GTP-Binding Proteins in DR

Apart from the classical mechanisms mentioned above, a few studies have recently demonstrated that small GTP-binding proteins play a crucial role in the pathogenesis of DR; these proteins are one of the biological switches of cellular processes. The family of small GTP-binding proteins is one of the regulators of the signaling cascade triggered by oxidative stress. GTP-binding proteins belong to a superfamily consisting of more than 100 members, which range from 20 to 40 kDa in size; there are five major families: Ras, Rho, Rab, Sar1/Arf, and Ran, which regulate processes such as cellular proliferation, survival, and differentiation [68]. These cellular processes cycle between GTP-bound active and GDP-bound inactive states. The Ras and Rho families are regarded as particularly important in the development of DR.

**Figure 5:**
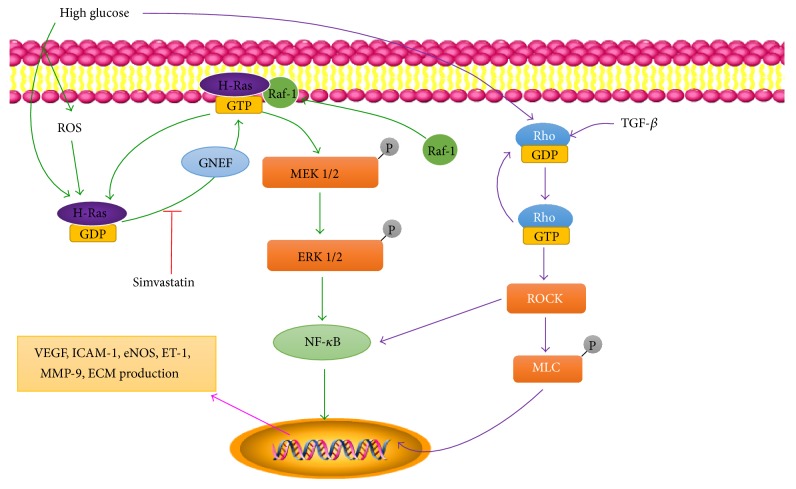
The mechanisms of Ras and Rho proteins in DR. High glucose activates H-Ras via ROS and/or other classical pathways, which contains two steps: H-Ras translocates from the cytosol to the membrane and H-Ras binds to GTP. The former can be inhibited by simvastatin; the latter can be activated by guanine nucleotide exchange factor (GNEF). After that, Raf-1 also can be activated by translocation to the membrane and binding with H-Ras, thus initiating the Ras/Raf/MEK/ERK pathway. On the other hand, Rho can be activated by high glucose and elevate the level of TGF-*β*, thus promoting the Rho/ROCK pathway. Both of these pathways lead to increased levels of various cytokines involved in DR. ECM, extracellular matrix; eNOS, endothelial nitric oxide synthase; ET-1, endothelin-1; ERK 1/2, extracellular signal regulated kinases 1/2; ICAM-1, intercellular adhesion molecule-1; GNEF, guanine nucleotide exchange factor; MEK 1/2, mitogen-activated protein kinase kinase 1/2; MLC, myosin light chain; MMP-9, matrix metalloproteinase-9; NF-*κ*B, nuclear factor-*κ*B; ROCK, Rho-kinase; TGF-*β*, transforming growth factor-*β*; VEGF, vascular endothelial growth factor.

The Ras family is a group of low-molecular weight GTP-binding proteins that are involved in cellular signal transduction; the family includes the three highly homologous proteins H-, K-, and N-Ras [69]. It is well known that diabetes increases oxidative stress and that ROS can influence the interactions between H-Ras and its several effector proteins [70]. The most important effector protein is Raf-1, a threonine/serine kinase. The Ras/Raf complex can, in turn, induce intracellular oxidative stress [71]. It has been demonstrated that the activation of retinal H-Ras in diabetes is mediated by its translocation to the plasma membrane, which can be prevented by simvastatin, an inhibitor that blocks the membrane translocation of H-Ras [72]. Evidence suggests that activated H-Ras produces more ROS, probably through activating Nox [73]. Moreover, H-Ras activation is thought to be involved in cell migration, proliferation, and differentiation, which leads to neovascularization [74]. Previous studies have indicated that the expression levels of H-Ras and its effector protein Raf-1 are increased in the retinas of diabetic animal models, suggesting that H-Ras is involved in retinal capillary apoptosis induced by high-glucose levels; furthermore, inhibitors of retinal H-Ras can prevent the development of DR [75, 76]. Several cell regulatory networks cannot be separated from the Ras/Raf/MEK/ERK pathway, which controls a wide range of downstream targets that regulate gene expression and transcription factors (Figure 5) [69]. There is also evidence that the Raf/MEK/ERK cascade can be activated by ROS, contributing to cellular dysfunction and/or apoptosis [77]. Matrix metalloproteinase-9 (MMP-9), the most complex member of the MMP family, is thought to be involved in vascular permeability and capillary apoptosis in DR owing to its damage to the tight junction complex and activation of caspase-3, respectively [78]. Diabetes causes the activation of MMPs in various tissues, and MMP-9 activity is increased in the retinas of patients and animal models with DR [78, 79]. However, MMP-9 expression is regulated by H-Ras. In rat liver epithelial cells, H-Ras activation can regulate MMP-9 expression [80], and overexpression of H-Ras in human fibroblasts is associated with the upregulation of MMP-9 [81]. Furthermore, MMP-9 activation is decreased following treatment with an H-Ras inhibitor [78]. This suggests that MMP-9 activation is regulated by H-Ras and that MMP-9 appears to be downstream of H-Ras. However, the exact mechanism remains unclear.

The Rho family and its target protein Rho-kinase (ROCK) are involved in cell migration, adhesion, proliferation, and apoptosis [68, 82]. The Rho/ROCK pathway is activated in retinal microvessels during diabetes and affects the expression and function of adhesion molecules, such as ICAM-1, leading to leukocyte adhesion to the microvasculature [82]. In endothelial cells, Rho/ROCK signaling activation is also involved in VEGF-induced migration and angiogenesis [83]. Moreover, it is well known that transforming growth factor-*β* (TGF-*β*) is overexpressed in the vitreous of patients with proliferative vitreoretinal disease and that it contributes to the cicatricial contraction of the membrane. Specifically, TGF-*β*2, the main isoform in the posterior segment of the eye, activates Rho, leading to myosin light chain phosphorylation in hyalocytes [84], which is associated with actin-myosin interaction to form stress fibers and cell contraction [85, 86]. This evidence shows that the activation of ROCK is important in the cicatricial contraction of the proliferative membrane in DR patients.

### 4.4. Epigenetic Modifications in the Oxidative Stress-Related Pathogenesis of DR

It is well known that the pathogenesis of DR continues after the termination of a high-glucose insult, a phenomenon known as “metabolic memory.” It has been suggested that epigenetic modifications are one of the major factors that contribute to the development of DR. Environmental factors, lifestyle, disease progression, and age influence epigenetic changes; DNA methylation, histone modification, and the effects of miRNAs and sirtuin proteins (SIRTs) are considered the major epigenetic modifications.

#### 4.4.1. DNA Methylation

DNA is not a fixed entity; it can modify its properties to adapt to environmental changes. The methylation of cytosine forms 5-methylated cytosine, which alters the chromatin structure by changing protein-DNA interactions; it is considered to be one of the major epigenetic modifications [87]. Normally, CpG islands, which are enriched in the promoter regions, comprise unmethylated cytosines. This guarantees the active expression of genes [88]. In experimental studies, hyperglycemic induction is associated with hypermethylation or hypomethylation of the promoter region in some genes that play critical roles in DR development. For example, research shows that a hyperglycemic milieu can alter the methylation status of the MMP-9 promoter, and the regulation of retinal MMP-9 promoter hypomethylation can prevent mitochondrial damage and the development of DR [89]. Generally, two classes of enzymes are involved in DNA methylation: DNA methyltransferases (DNMTs) and ten-eleven translocation enzymes. The former can transfer a methyl group from S-adenosyl methionine to cytosine [90]; the latter oxidize 5-methylated cytosine to produce 5-hydroxymethyl cytosine, which is easily oxidized to generate 5-formylcytosine and 5-carboxylcytosine [91]. DNMT activity is increased and the expression of DNMT1 is elevated in diabetic retinas and their capillary cells [16]. In the retinas of rats with STZ-induced diabetes, the promoter region of DNA polymerase gamma is hypermethylated, which is sustained even after 3 months of good glycemic control [92]. High levels of glucose also activate ten-eleven translocation enzymes in zebrafish, leading to genome-wide demethylation and aberrant gene expression [93]. Therefore, DNA methylation is important in the process of DR and metabolic memory.

#### 4.4.2. Histone Modification

Histone modification is another influential factor in diabetes. Chromatin comprises nucleic acids and histones, which are proteins that can regulate the structure of DNA and the expression of various genes. There are five major histone families: H1/H5, H2A, H2B, H3, and H4. Histones constitute the basic unit, the nucleosome, of chromatin and act as a spool around which the DNA is wound. The N-terminal sequences of histones can be modified by acetylation, phosphorylation, methylation, ubiquitination, or sumoylation [87]. Acetylation and methylation are the most common modifications of histones. The acetylation of histones at lysine residues generally occurs with transcriptionally active genes, whereas the methylation of lysine is related to gene activation or repression [94, 95]. Two groups of enzymes are involved in acetylation: histone acetyltransferases and histone deacetylases, which can add or remove acetyl groups, respectively. Histone acetyltransferase overactivity results in hyperacetylation, which can facilitate the binding of transcription factors and RNA polymerases to the DNA template [96]. There is evidence that, in rats with STZ-induced diabetes, the activity and transcripts of histone deacetylases are increased, and histone acetyltransferase activity is reduced, which leads to the reduced acetylation of histone H3 [97]. Another research has shown that modification of histone H3 at lysine 9 (H3K9) at the proximal Cox2 promoter bearing the NF-*κ*B binding site is involved in thioredoxin-interacting protein-mediated inflammation in high-glucose cultured retinal endothelial cells [98]. Moreover, transient hyperglycemia can induce upregulation of NF-*κ*B subunit p65, which acts as an inflammatory mediator in diabetes, via active histone H3 lysine 4 monomethylation at the promoter region [99]. However, hypomethylation of H3K9 is also observed in diabetes, and this frees up the lysine 9 of H3K9 for acetylation, facilitating the recruitment of NF-*κ*B [100]. Furthermore, the promoter and enhancer regions of superoxide dismutase-2 (SOD-2) change in diabetes [101], and histone lysine acylation and methylation in monocytes at inflammation- and diabetes-related gene loci can be induced by hyperglycemia [102, 103]. Both hyperglycemia and hydrogen peroxide treatment increase the expression of coactivator-associated arginine methyltransferase 1 via histone 3 arginine 17 asymmetric demethylation in RPE cells, suggesting that oxidative stress-mediated cell damage is associated with histone modification [104, 105].

#### 4.4.3. MicroRNAs (miRNAs)

MicroRNAs (miRNAs) are small, single-stranded, noncoding RNA molecules. They contain 20–24 nucleotides and regulate genes by binding to the target sequences in the 3′-untranslated regions of messenger RNAs [106]. Gene expression can be controlled by miRNA via either degradation or translation repression of messenger RNA, and gene regulation is involved in inflammation, cellular metabolism, angiogenesis, and apoptosis [107]. Circulating miRNA is altered in diabetic patients, and the expression of miRNAs can be a predictive factor for the progression of diabetes [108]. Recent research has shown that miRNA-200b is upregulated in diabetic mouse retinas. This increases the expression of oxidation resistance-1, which prevents oxidative stress [109]; therefore, miRNA-200b has a protective role in DR. Moreover, the expression of miRNA-26b-5p increases after exposure to high levels of glucose [110], and miRNA-26b-5p facilitates cardiomyocyte apoptosis by increasing the production of ROS [111], suggesting its contribution to endothelial apoptosis in DR. Other experiments have provided evidence that oxidative stress regulates SIRT1, which has significant antioxidative and anti-inflammatory effects through miRNA; several miRNAs such as miRNA-217, miRNA-181, miRNA-138, and miRNA-199 downregulate SIRT1 in various cells and tissue types [112]. These studies suggest that miRNAs play an important role in regulating gene expression in DR.

#### 4.4.4. SIRTs

SIRTs are homologs of silent information regulator 2 in higher eukaryotic organisms. They participate in the epigenetic modifications of histone and nonhistone protein deacetylation, which contribute to the mechanisms of vascular dysfunction related to aging, cardiovascular disease, diabetes, and other metabolic diseases [113, 114]. Mammals have seven SIRTs (SIRT1–7) that all depend on NAD^+^ cofactor. SIRT1 and SIRT3 are closely correlated with diabetes and its complications. This is particularly true of SIRT1 [115, 116], which regulates oxidative stress response and RPE cell apoptosis via the deacetylation of cytoplasmic p53 [117]. A recent study has shown that SIRT1 activity decreases in diabetic retinas [118]. Moreover, SIRT1 overexpression protects pancreatic *β*-cells by inhibiting the NF-*κ*B pathway through deacetylation of p65 [119]. In cultured mammalian cells, SIRT1 is activated in response to energy stress and increased oxidative stress. Evidence also shows that SIRT1 is closely related to redox modulation because its activity depends on intracellular NAD^+^[120]. Furthermore, exendin-4 reduces the number of retinal cells and the production of ROS by upregulating the expression of SIRT1 and SIRT3 in the retinas of early-stage diabetic rats [121].

## 5. Antioxidant Treatments for DR

In the previous sections, we have demonstrated that oxidative stress plays a crucial role in the development of DR. Therefore, it is postulated that antioxidants inhibit abnormal metabolism and slow DR progression by inhibiting the production of ROS, neutralizing free radicals, or augmenting the antioxidant defense system. Therefore, these factors are targets in the treatment of DR.

### 5.1. Vitamins

Vitamins C and E can protect against the development of DR by scavenging free radicals, reducing the production of ROS, and preventing lipid peroxidation [122, 123]. Therefore, supplementation with vitamins C and E is thought to be helpful in the treatment of DR. Vitamin C can ameliorate endothelial dysfunction in diabetes and decrease leukocyte adhesion in retinal vessels in diabetic rats [123]. Vitamin E inhibits the PKC pathway in diabetes; the PKC pathway induces a decrease of retinal blood flow, resulting in retinopathy [124]. Recently, vitamin D has attracted interest as being important in the development of DR. Research shows that vitamin D deficiency is very common in type II diabetes, and there is an increased risk of retinopathy in diabetic patients with severe vitamin D deficiency [125]. Further research has demonstrated that vitamin D can inhibit inflammation as well as the expression of VEGF and TGF-*β*1 in retinal tissues, thereby protecting the retina from neovascularization and cell proliferation [126].

### 5.2. Green Tea

Green tea, which is rich in polyphenols, is thought to have potent antioxidative, anti-inflammatory, and anticarcinogenic properties [127, 128]. Green tea supplementation in diabetic rats increases the level of GSH and the activities of SOD and catalase, reduces the expression of VEGF and TNF-*α*, and protects retinal vessels from angiogenesis and retinal endothelial cells from apoptosis [128]. These results indicate the possible mechanisms of the beneficial effects of green tea in the treatment of DR.

### 5.3. *α*-Lipoic Acid (ALA)

ALA is a powerful antioxidant that is necessary for the function of several enzymes involved in oxidative metabolism [129]. It can reduce the amounts of oxidized forms of other antioxidants such as GSH and vitamins C and E and can modulate several of the signal transduction pathways involved in vascular damage, such as the NF-*κ*B pathway [130]. It has been shown that ALA supplementation in diabetic rats over 30 weeks can prevent damage to retinal vessels, including pericyte dropout, and ALA can also inhibit the expression of Ang-2, VEGF, and AGE receptors, which are related to pericyte dropout, neovascularization, and the activation of NF-*κ*B [131].

### 5.4. Benfotiamine

Benfotiamine, a lipophilic analogue of thiamine monophosphate, can protect vascular cells from the metabolic damage induced by hyperglycemia. In diabetic animals, benfotiamine has been shown to inhibit three major ROS production pathways (the hexosamine, PKC, and AGE pathways) and can reduce the activation of NF-*κ*B by exciting transketolase [132]. There is also evidence that benfotiamine can protect retinal pericytes from apoptosis via normalizing the Bcl-2/Bax ratio [133]. Therefore, benfotiamine might provide an alternative therapy for DR.

### 5.5. Poly (ADP-Ribose) Polymerase (PARP) Inhibitors

PARP is one of the downstream oxidant injury mediators. It is involved in retinal cell apoptosis and the regulation of NF-*κ*B activation [134]. PARP can increase the expression of vasoactive factors and the production of extracellular matrix proteins in diabetic animals, and the genetic and chemical blockade of PARP can normalize these changes [135]. Administration of PJ-34, a potent PARP inhibitor, decreases the activation of NF-*κ*B and the transcription of NF-*κ*B-dependent genes such as ICAM-1 in diabetic rats [136]. The evidence supports the value of PARP as a therapeutic target for delaying the development of DR.

### 5.6. SOD

SOD, the first line of physiological defense against oxidative stress, also contributes to the development of DR. The overexpression of manganese SOD can decrease the hyperglycemia-induced expression of VEGF mRNA and protein in endothelial cells, and the expression of VEGF and fibronectin in STZ-induced DR models can also be reduced [137]. Moreover, overexpression of mitochondrial SOD in hemizygous transgenic mice can prevent diabetic retinal oxidative stress and mitochondrial dysfunction [138]. Tempol, a SOD mimetic, can scavenge O_2_^•−^, reduce the amount of NO end-products, and increase GSH biosynthesis, thereby normalizing the oxidative/nitrosative status of DR. Furthermore, decreased copper-zinc SOD expression/activity in treated diabetic rats has been observed [139].

### 5.7. Other Antioxidants

Other antioxidants also have beneficial effects on the development of DR. For example, zinc, an important nutrient, is involved in oxidative stress, apoptosis, and aging. Zinc can prevent the increase of lipid peroxidation induced by diabetes in the retina and can reduce GSH levels in diabetic rats [140]. Moreover, Trolox (6-hydroxy-2,5,7,8-tetramethylchroman-2-carboxylic acid) and N-acetyl-L-cysteine prevent glycated albumin-induced cell death in pericytes [141]. Nicanartine, an antioxidant with cholesterol-lowering properties, can partially inhibit pericyte loss in diabetic rats [142]. Curcumin, a traditional herb extract, demonstrates significant antioxidant activity by downregulating hypoxia-inducible factor 1 and preventing angiogenesis induced by hypoxia [143]. Curcumin supplementation can inhibit the expression of VEGF in DR rats [144]. Furthermore, *β*-carotene, taurine, lutein, caffeic acid phenethyl ester, cannabidiol, and 8-hydroxy-N,N-dipropyl-2-aminotetralin also have some antioxidant properties [145].

### 5.8. Nrf2-Related Treatments

In the previous sections, we have described the role of Nrf2-signaling pathways in DR and inflammation [58]. Sulforaphane, an Nrf2 inducer, can protect the RPE against oxidative injury [146]. Hemin is another potential treatment. In diabetic rats, the levels of heme oxygenase-1, SOD-1, and Bcl-2 increase, whereas the expressions levels of hypoxia-inducible factor 1 and VEGF decrease following treatment with hemin. The activation of Nrf2 nuclear translocation contributes to these results [147]. Mycophenolate mofetil (MMF) is also considered an effective drug. Evidence shows that MMF facilitates Nrf2 nuclear translocation and conserves antioxidant enzymes in diabetic nephropathy [148]. Moreover, zinc, oltipraz, and dimethyl fumarate have different effects in diabetes via the Nrf2 pathway [149–151].

### 5.9. Epigenetic Modification-Related Treatments

As mentioned above, DNA methylation, histone modification, and miRNAs are three major epigenetic factors that are potential targets for the treatment of diabetes. DNA methylation can be repressed by nucleoside analogues because they inhibit DNMTs. Moreover, DNMT inhibitors, including 5-azacytidine (azacitidine; Vidaza) and 5-aza-20-deoxycytidine (decitabine; Dacogen) for myeloid cancers and cutaneous T cell lymphoma, have been recommended by the US Food and Drug Administration [87]. The regulation of histone modification also has a large effect. For instance, epigallocatechin-3-gallate, a strong histone acetyltransferases inhibitor, can block the activation of p65 acetylation-dependent NF-*κ*B [152]. Curcumin, genistein, and resveratrol also have an influence on histone modification [153]. Resveratrol has a protective role against inflammation because it controls the expression of several miRNAs such as miRNA-21, miRNA-155, and miRNA-663 [154]. For miRNA, double-stranded miRNA mimics and anti-RNA antisense oligodeoxyribonucleotides are major molecules that have been investigated for targeting specific miRNAs [9]. Metformin, which is involved in the SIRT1/LKB1/AMPK pathway, suppresses hyperglycemia stress “memory” in diabetic rats [155]. Another research has shown that cocoa enriched with polyphenol improves the retinal SIRT1 pathway and protects the retina from diabetic insult [156]. Moreover, resveratrol and fenofibrate also have a protective effect on DR via SIRT1-related pathways [118, 157].

## 6. Conclusions

DR is a leading cause of blindness in working-age adults, and oxidative stress plays an important role in its development. In this review, we have demonstrated that hyperglycemia induces a series of metabolic abnormalities in the retina by producing ROS and subsequently initiating oxidative stress. This can in turn activate these abnormal metabolic pathways to produce more ROS, thereby creating a vicious cycle. Generally, oxidative stress causes retinal damage by inducing endothelial cell dysfunction, pericyte apoptosis, and angiogenesis. The polyol, AGE, PKC, hexosamine, and Nrf2-related pathways, GTP-binding proteins, and epigenetic modifications are all involved in the pathological process of DR. Therefore, inhibitors of these pathways may protect the retina from damage induced by high levels of glucose. Antioxidants are beneficial for DR because they reduce ROS production, neutralize free peroxynitrite, or augment the antioxidant defense system. Furthermore, herbal extracts have received increasing attention for treating DR in recent years, although the mechanisms underlying their mode of action require verification. At present, most of the evidence has been provided by animal experiments, and the clinical therapeutic effects are still not very clear. Thus, more work is needed in the future.
